# Stability Mechanism of Two Soybean Protein-Phosphatidylcholine Nanoemulsion Preparation Methods from a Structural Perspective: A Raman Spectroscopy Analysis

**DOI:** 10.1038/s41598-019-43439-5

**Published:** 2019-05-06

**Authors:** Ying Zhu, Yang Li, Changling Wu, Fei Teng, Baokun Qi, Xiaonan Zhang, Linyi Zhou, Guoping Yu, Huan Wang, Shuang Zhang, Zhongjiang Wang, Lianzhou Jiang

**Affiliations:** 1 College of Food Science, Key Laboratory of Soybean Biology in Chinese Ministry of Education, Northeast Agricultural University, Harbin, 150030 China; 2 Institute of Food Industry Research in Harbin, Harbin, 150030 China; 3 National Research Center of Soybean Engineering and Technology, Harbin, 150030 China

**Keywords:** Phosphoproteins, Raman spectroscopy, Phosphoproteins, Raman spectroscopy

## Abstract

Ultrasound treatment and high-pressure homogenization were used to prepare soybean protein (SP)-phosphatidylcholine (PC) nanoemulsions in this study. Nanoemulsions prepared by high-pressure homogenization were more stable. The structural changes of SP and PC under ultrasound treatment and high-pressure homogenization treatment were investigated by Raman spectroscopy. It could be concluded that ultrasound and high-pressure homogenization treatments increased both the content of α-helix and unordered structure but decreased that of β-structures of SP, while the interaction between SP and PC decreased α-helix content and also reduced unordered structure and β-sheet structure. Ultrasound treatment and high-pressure homogenization exposed more tryptophan and tyrosine residues to promote hydrophobic interaction between SP and PC, which was beneficial for stabilizing the nanoemulsion. The SP-PC interaction exerted a more significant effect on side chain structure than those observed under ultrasound treatment and high-pressure homogenization. The dominant *g-g-t* vibrational mode of the disulfide bond of soybean protein was not appreciably changed by the two preparations. High-pressure homogenization increased the disorder of lipid chains of PC, promoting SP-PC interaction and thereby increasing the stability of the nanoemulsion. The structural change provided a theoretical basis for preparation of two nanoemulsions.

## Introduction

Soybean protein (SP) has received increasing attention as a highly nutritious food ingredient with good solubility, emulsibility, foamability, gel foaming properties, hydratability and water binding capacity^1^. However, emulsions prepared by soybean protein were sensitive and sometimes unstable, which limited its applications in food production^2^. Phosphatidylcholines (PC) are a class of phospholipids representing the primary components of biological membranes. As a healthy and amphoteric surfactant, PC has been reported to interact with protein to modify the protein structure and the interfacial properties of a subsequent emulsion, thus enhancing the protein emulsifying capacity and affecting the microencapsulation properties of proteins^3–6^. According to Ohtsuru *et al*., PC was associated with soybean protein through two types of interaction: a hydrophobic interaction between a PC molecule and the hydrophobic regions of the protein, and the binding of the PC lamellae to the protein surface. The hydrophobic interactions were the main forces maintaining the stability of SP-PC complexes, followed by hydrogen bonding^7^. It was reported that soybean protein isolates and phospholipids present specific surface properties with synergistic or antagonistic effects on emulsion stability^6^. Phospholipids, primarily composed of PC, prevented heat-induced protein aggregation and increased the heat stability of dairy emulsions^8^. Furthermore, Kasinos *et al*. reported that the heat stability of solutions and emulsions containing whey protein were significantly improved through the addition of sunflower phospholipids^9^.

Nanoemulsions have smaller droplet sizes (average droplet diameter less than 200 nm) and higher stability, enabling widespread use in foods and beverages^10^. Ultrasound treatment and high-pressure homogenization were widely used to prepare nanoemulsions and improve the functional properties of proteins. For example, the emulsifying properties of soybean protein isolate stabilized by phospholipid were improved by ultrasound treatments^11^. Ultrasound treatment has been reported to cause partial unfolding of soybean proteins and the reduction of intermolecular interactions within the proteins^12^. The high-pressure homogenization method was used to prepare stable O/W emulsions stabilized by phospholipids and proteins^13–15^. Comas *et al*. discovered that phospholipids could enhance the stability of the SP emulsion prepared by high-pressure homogenization^3^. However, the stability mechanism of SP-PC nanoemulsions prepared by high-pressure homogenization and ultrasound treatment remains unclear.

Raman spectroscopy is a novel technology which is unaffected by the presence of water. It can be easily applied and represents an excellent approach to protein surveillance within solid or aqueous samples and for detecting the interaction between samples, providing information on the protein structure, the environment of some side chains and the local conformations of disulfide bonds^16–18^. As such, this study aimed at analyzing the interaction and structural changes of SP-PC complexes induced by ultrasound treatment and high-pressure homogenization using Raman spectroscopy to provide a better understanding of the preparation and properties of SP-PC nanoemulsions.

## Results and Discussion

### Characteristics of SP-PC nanoemulsions prepared by two methods

**Figure 1:**
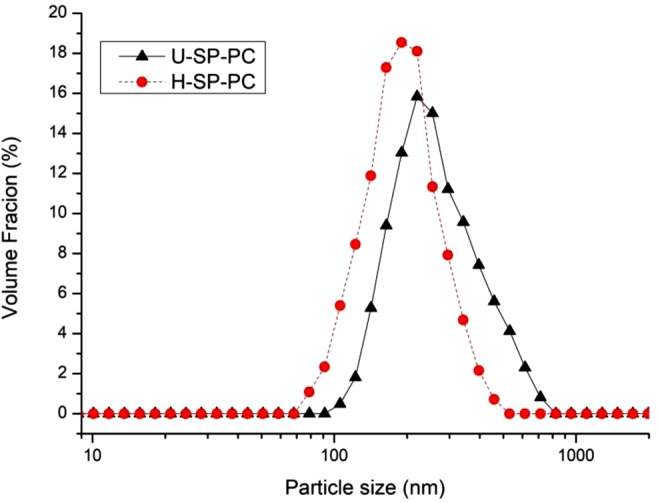
Particle size distribution of the nanoemulsions prepared by ultrasound and high-pressure homogenization treatments.

Many properties of the nanoemulsion, such as stability, structural characteristics and rheology, were related to particle size distribution and PDI. According to Stokes’ law, the velocity of droplet motion was proportional to the square of its radius^19^. Nanoemulsions exhibited better stability against droplet flocculation and coalescence with smaller particle sizes because they decreased the range of the attractive forces acting between the droplets^20^. The extremely small droplet size in nanoemulsions provided them with various underlying benefits over common emulsions: high optical clarity, good stability to gravitational separation and particle aggregation, and enhanced bioavailability^20^. The particle sizes of SP-PC nanoemulsions prepared by ultrasound treatment and high-pressure homogenization are shown in Table 1 and Fig. 1. The two treatments both exhibited a unimodal particle size distribution, while the average particle size of SP-PC nanoemulsions prepared by ultrasound treatment was 282.4 nm, which was significantly larger than that of high-pressure homogenization^21^. Seidmahdi *et al*.^22^ found that the nanoemulsion particle size as-prepared by high-pressure homogenization was 0.1 μm, while the ultrasound-prepared nanoemulsion particles were larger. In addition, it could be seen that the mean particle size of nanoemulsions prepared by ultrasound treatment increased from 282.4 nm to 309.8 nm during 30 days of storage, while the mean particle size of nanoemulsions prepared by homogenization slightly increased from 217.4 nm to 223.8 nm. It could be concluded that SP-PC nanoemulsions prepared by high-pressure homogenization were more stable.

**Table 1:** Characteristics of SP-PC nanoemulsions prepared by ultrasound and high-pressure homogenization treatments.

Sample	Storage time (d)	U-SP-PC nanoemulsion	H-SP-PC nanoemulsion
PDI	0	0.21 ± 0.01^b^	0.20 ± 0.01^a^
	15	0.27 ± 0.02^a^	0.21 ± 0.02^a^
	30	0.27 ± 0.03^a^	0.23 ± 0.01^a^
Mean Particle size (nm)	0	282.4 ± 2.5^c^	217.4 ± 1.7^b^
	15	291.6 ± 4.7^b^	220.5 ± 2.1^a^
	30	309.8 ± 3.6^a^	223.8 ± 4.1^a^
ζ-Potential value (mV)	0	−32.40 ± 0.28^a^	−34.60 ± 0.30^a^
	15	−31.40 ± 0.19^b^	−34.40 ± 0.11^a^
	30	−30.40 ± 0.15^c^	−33.80 ± 0.28^b^
TSI	0	3.10 ± 0.03^c^	3.02 ± 0.03^c^
	15	3.22 ± 0.01^b^	3.11 ± 0.03^b^
	30	3.45 ± 0.02^a^	3.18 ± 0.03^a^

Different letters^(a, b, c)^ in the same column indicate significant differences (P < 0.05).

PDI represents the degree of uniformity of the distribution size of nanoemulsions, and smaller PDI value indicated more homogeneous particle size distribution of nanoemulsions. As depicted in Table 1, the nanoemulsions prepared by the two treatments showed similar PDI values. Silva *et al*.^23^ found that ultrasound and high-pressure homogenization promoted nanoemulsion dispersion and avoided recoalescence phenomena, which consequently proved that the two treatments each decreased the PDI of nanoemulsions. No significant change was observed regarding PDI of nanoemulsions prepared by homogenization during storage, but PDI of nanoemulsions prepared by ultrasound treatment increased from 0.21 to 0.27, which might be related to the increased particle size.

The ζ-potential value was a measure of the electrostatic repulsion of the droplet, and the higher its absolute value, the better its stability. When the absolute ζ-potential value was greater than 30 mV, nanoemulsions could be stabilized by strong interparticle electrostatic repulsion. The absolute ζ-potential values of two nanoemulsions were higher than 30 mV, which suggested that the two nanoemulsions possessed greater ability to inhibit coalescence^24^. Higher absolute ζ-potential values represented stronger electrostatic repulsion between the nanoemulsion droplets. The absolute ζ-potential value of the nanoemulsion prepared by high-pressure homogenization was higher than that obtained by ultrasound treatment, and the reduction of the absolute ζ-potential value of the nanoemulsion prepared by high-pressure homogenization during storage was lower, both of which suggested that homogenization produced a more stable nanoemulsion than ultrasound in this study.

Turbiscan stability index (TSI), which had previously been used to evaluate the stability of colloidal dispersions, was used in the present study to investigate the physical stability of nanoemulsions. Nanoemulsions with lower TSI value were more stable and able to prevent coalescence and flocculation^25^. The TSI values of SP-PC nanoemulsions prepared by high-pressure homogenization were lower than those of ultrasound treatment, which might be due to the relation between TSI and electrostatic interaction and hydrodynamic interaction at the interface of nanoemulsion particles. TSI of nanoemulsions prepared by ultrasound treatment increased significantly from 3.10 to 3.45, while TSI of those prepared by homogenization increased from 3.02 to 3.18, which confirmed that the storage stability of nanoemulsions prepared by homogenization was superior. It could be concluded from the abovementioned results that nanoemulsions prepared by high-pressure homogenization were more stable.

### Microscopy measurement

**Figure 2:**
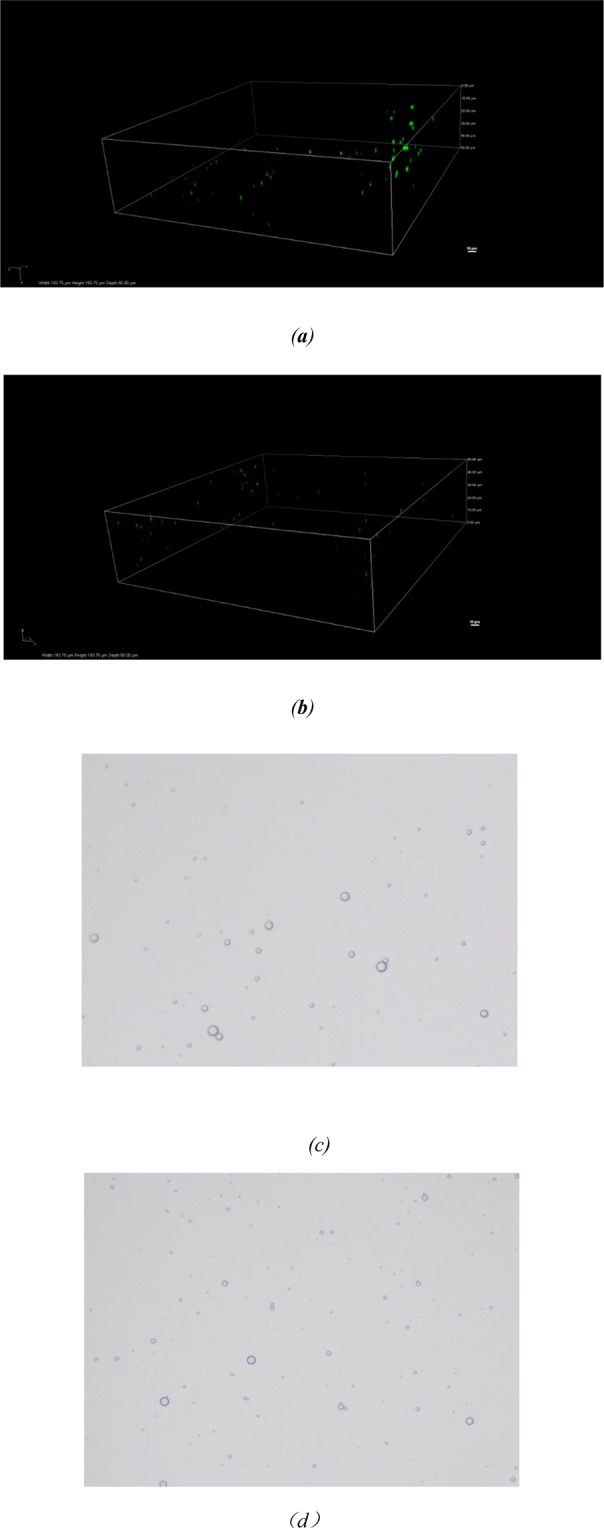
Different microscopy of nanoemulsions under different preparation methods 3D Confocal laser scanning microscopy: (**a**) the nanoemulsion prepared by ultrasound; (**b**) the nanoemulsion prepared by high-pressure homogenization; Optical microscopy: (**c**) the nanoemulsion prepared by ultrasound; (**d**) the nanoemulsion prepared by high-pressure homogenization.

3D confocal laser scanning microscopy and optical microscopy represented the new technique to detect the distributions of protein and oil droplets due to their small droplet size^26^. As shown in Fig. 2, it could be seen that the droplets corresponding to two nanoemulsions presented spherical morphology and that SP was adsorbed at the interface of nanoemulsions, showing a “core-shell” structure. The green fluorescence periphery in CLSM micrographs represented the protein portion, which indicated the protein located on the surface of nanoemulsions^27^. Moreover, the nanoemulsion droplets remained dispersed by microscopy images, which could be attributed to the two preparation methods. Ultrasound treatment and high-pressure homogenization both prepared stable nanoemulsions. In comparison, the particle sizes of nanoemulsions prepared by homogenization were smaller, which was in agreement with the result obtained for the particle size distributions of the emulsions.

### Raman spectroscopy

**Figure 3:**
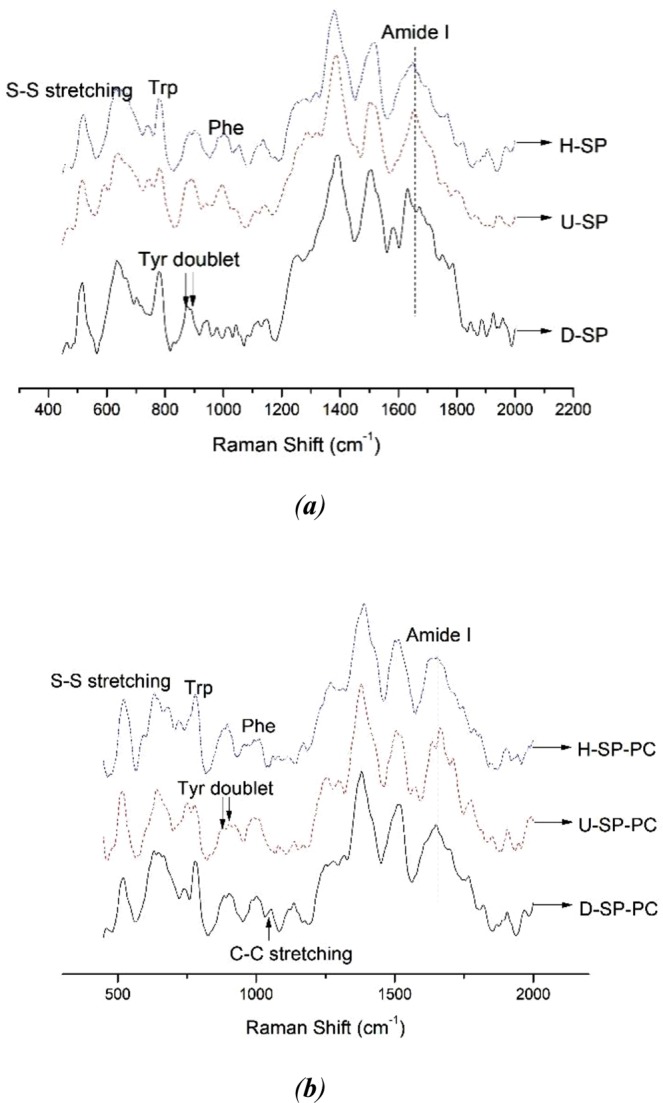
Raman spectra of nanoemulsions after different treatments: (**a**) the SP under ultrasound and high pressure homogenization; (**b**) the SP-PC nanoemulsions under ultrasound and high-pressure homogenization.

Modifications to the Raman bands of protein chemical groups primarily conferred information regarding changes in the secondary structure of proteins (amide conformation region, C-C stretching vibration) and modifications in local environments (tryptophan residues, tyrosyl doublet, aliphatic amino acids bands). The typical Raman spectra in the 400–2000 cm^−1^ region for SP and SP-PC with different treatments, respectively, are shown in Fig. 3. The assignments of some major peaks were made based on previous works listed in Table 2 ^16,28^.

**Table 2:** Assignment of Raman modes useful in the interpretation of protein structure.

Frequency (cm^−1^)	Assignment
500–550	Disulfide bond
620–640	Phe
644	Tyr
720	C-N stretching
760	Trp
830	Tyr ν-ring
850	Tyr ν-ring
940	νCC (α-helix)
1003	Phe ν-ring
1062	C-C stretching
1250	Amide III (β-sheets, random coil)
1273	Amide III (α-helix)
1309	Amide III (α-helix)
1321	Trp ν-ring
1340	δCH
1360	Trp ν-ring
1450	δasCH_3_, δCH_2_, δCH
1645–1690	Amide I
1748	C=O stretching
2850	C-H stretching
2880	C-H stretching

#### Changes in secondary structure (amide I band)

The most useful Raman band for determining the secondary structure of SP was the amide I band from 1600 to 1690 cm^−1^ ^16,29^. Baseline correction in the amide I band was performed to find the extreme points of the band profile along the wavenumber axis^30^. To avoid noise in the spectra, Savitzky Golay smoothing was performed with a second order polynomial and 5 points of window included (Origin 8.5 software), then Gaussian fitting of the amide I bands for the different samples was conducted. The band positions inside the profile of the amide I band were determined using the second derivative. The peak positions of the amide I band were located as follows: α-helix, 1645–1660 cm^−1^; β-sheet, 1665–1680 cm^−1^; β-turn, 1680–1690 cm^−1^; unordered structure, 1660–1670 cm^−1^. The compositions of the secondary structure of SP with different treatments were listed in Table 3.

**Table 3:** Percentages of protein secondary structure of SP with different treatments.

Sample	α-helix	β-sheet	β-turn	Random coil
D-SP	30.59 ± 0.30^e^	24.28 ± 0.20^c^	16.76 ± 0.04^a^	28.37 ± 0.20^f^
D-SP-PC	29.40 ± 0.20 ^f^	28.07 ± 0.08^a^	10.00 ± 0.10^e^	32.53 ± 0.19^b^
U-SP	35.25 ± 0.08^b^	20.68 ± 0.10^e^	13.56 ± 0.10^b^	30.50 ± 0.30^d^
U-SP-PC	32.31 ± 0.20^c^	24.62 ± 0.20^b^	8.85 ± 0.06^f^	34.23 ± 0.26^a^
H-SP	38.64 ± 0.06^a^	21.59 ± 0.16^d^	10.70 ± 0.05^d^	29.07 ± 0.18^e^
H-SP-PC	31.34 ± 0.10^d^	24.88 ± 0.03^b^	12.41 ± 0.08^c^	31.37 ± 0.08^c^

Different letters^(a, b, c, d, e, f)^ in the same column indicate significant differences (*P* < 0.05).

It could be deduced from Fig. 3 that the position of the maximum wavenumber was located approximately 1650–1670 cm^−1^, which suggested that α-helix and unordered structure were the primary structures represented in soy protein. Accordingly, as depicted in Table 3, the native soybean protein exhibited 30.59% α-helix, 24.28% β-sheet, 16.76% β-turn and 28.37% unordered structure. In comparison, ultrasound treatment and high-pressure homogenization treatment both induced an increase in the content of α-helix and unordered structure while decreasing the percentage of β-sheet and β-turn structures in SP.

Ultrasound treatment was reported to unfold the structure of the protein and broke the peptide bonds to decrease the content of β-sheets^31^. Similar changes had been previously reported: SPI treated by higher power ultrasound treatment (400 W and 600 W) resulted in an increase in the α-helix component and a decrease in β-sheets^32^. It had been reported that β-sheet structure was mainly maintained by hydrogen bonds and that ultrasound treatment might have weakened or destroyed hydrogen bonds between the carbonyl group and amide group from the neighboring amino acid on the peptide chain, decreasing the content of β-sheet structure^33,34^. Stathopulos *et al*. reported that the decreased content of β-sheet was related with the exposed residues, leading to an enhancement of the surface hydrophobicity of the protein, which increased the emulsion activity of the protein^35^.

Vivian *et al*. and Jing *et al*. concluded that ultrasound treatment and ultrahigh-pressure homogenization resulted in an increase in the α-helix component and a decrease in the β-sheet component of whey protein concentrate^36,37^. In this study, high-pressure homogenization increased the content of α-helix and unordered structure while decreasing the percentage of β-conformation in SP. Zhang *et al*.^21^ reported that high-pressure homogenization could break the emulsion droplets into smaller species to stabilize nanoemulsions and α-helix structures of soybean protein isolates by intense turbulence, vibration, cavitation and hydraulic shear. The increased unordered structure induced by high-pressure homogenization was related with the increased flexibility of protein structure, which could determine that the emulsifying properties and structures rearranged rapidly on the interface^38,39^. Thus, high-pressure homogenization and ultrasound treatment not only exerted effects on the secondary structures, but also influenced molecular interactions^40^.

In comparison, the SP-PC complexes had a lower percentage of α-helix and β-turn structure and a higher content of β-sheet and unordered structure, which suggested that hydrophobic interactions between SP-PC resulted in the decrease of α-helix and β-turn and the increase of β-sheet and unordered structure. Ohtsuru *et al*. observed that SP was changed by the influence of PC, especially that β-structure content was decreased while unordered structure was increased^41^. Furthermore, the SP in combination with PC resulted in improvements to the emulsification activity and stability^6,42^. Meanwhile, ultrasound treatment and high-pressure homogenization both increased the content of α-helix structure and decreased that of β-sheet structure in SP reacted with PC. Ultrasound treatment increased the content of random coil structures of SP for interaction with PC, which decreased during high-pressure homogenization, representing the critical reason for the difference in SP-PC nanoemulsion stability when prepared by ultrasound treatment as opposed to high-pressure homogenization.

#### Structural changes of side chain

Several Raman bands mainly provide information about protein tertiary structure and intermolecular interactions, including tryptophan (Trp) bands, tyrosine (Tyr) bands and aliphatic hydrophobic residues^43^. The decrease in the intensity of a band near the 760 cm^−1^ region was attributed to the exposure of Trp residues in the protein^44^. In comparison with SP, the normalized intensity of the Raman band of tryptophan residues in SP under ultrasound treatment generally decreased, which indicated that the Trp residue tended to become exposed under ultrasound treatment. Our previous work indicated that sonication would increase the hydrophobic areas of the proteins exposed to the surface of the molecules^45^. However, no significant differences in the normalized intensity of the Raman band of tryptophan residues was observed between SP and H-SP homogenization, which suggested that homogenization did not significantly alter the microenvironment of the Trp residue. Additionally, the intensity of the Raman band corresponding to the Trp of SP increased due to the interaction with PC. One possible reason for this could be that the hydrophobic residues in SP were reburied during the interaction with PC; another explanation is that SP might be linked with PC through the hydrophobic residues, increasing the Raman intensity of SP. The Raman intensity of Trp in SP-PC treated by ultrasound and high-pressure homogenization significantly increased, which further verified that the ultrasound and high-pressure homogenization enhanced the hydrophobic interaction of SP-PC, increasing the stability of nanoemulsions.

The bands located near 850 cm^−1^ and 830 cm^−1^ represent the Fermi doublet of tyrosine, known to be a good indicator or acceptor state of the tyrosine phenolic hydroxyl group^46^. The tyrosyl doublet ratio (*I*_850_/*I*_830_) was proposed as a means of determining whether the tyrosine residue was solvent-exposed or buried. If the intensity ratio *I*_850_/*I*_830_ was 2.5, the hydroxyl oxygen atom on the Tyr benzene ring was a strong hydrogen bond receptor. If the ratio of *I*_850_/*I*_830_ was 1.25, the hydroxyl oxygen atom on the Tyr benzene ring was the donor or acceptor of the hydrogen bond. If the ratio was 0.3, the hydroxyl oxygen atom on the benzene ring of Tyr was a donor of a strong hydrogen bond^47,48^. In this study, the ratio of *I*_850_/*I*_830_ of the tested protein was distributed between 1.01 and 1.06, indicating that the Tyr residues were exposed to the aqueous or polar microenvironment and acted as simultaneous acceptors and donors of moderate to weak hydrogen bonds. In comparison, ultrasound treatment and high-pressure homogenization significantly increased the tyrosyl doublet ratio, which suggested that the Tyr residue in soybean protein treated by ultrasound treatment and high-pressure homogenization tended to be exposed to provide more interaction sites for PC to stabilize the nanoemulsion. Furthermore, SP-PC interaction decreased the tyrosyl doublet ratio, further confirming that the SP and PC interaction site was located at a hydrophobic side chain group. As shown in Table 4, no significant differences were observed in Tyr doublet ratios of SP-PC complexes under different treatments, which indicated that ultrasound treatment and high-pressure homogenization did not significantly alter the microenvironment around tyrosyl residues in SP interacting with PC.

**Table 4:** Intensities of tryptophan band, tyrosyl doublet of soy protein under different treatments.

Sample	Trp (*I*_760_/*I*_1003_ cm^−1^)	Tyr doublet (*I*_850_/*I*_830_ cm^−1^)	CH (*I*_1450_/*I*_1003_ cm^−1^)
D-SP	1.03 ± 0.01^c^	1.01 ± 0.01^b^	1.32 ± 0.01^a^
D-SP-PC	1.05 ± 0.01^d^	0.99 ± 0.01^a^	1.37 ± 0.00^d^
U-SP	0.99 ± 0.00^b^	1.05 ± 0.01^c^	1.25 ± 0.01^b^
U-SP-PC	1.07 ± 0.01^c^	1.00 ± 0.00^b^	1.37 ± 0.01^d^
H-SP	1.04 ± 0.00^a^	1.06 ± 0.01^c^	1.17 ± 0.01^d^
H-SP-PC	1.08 ± 0.01^c^	1.00 ± 0.00^b^	1.38 ± 0.01^c^

Different letters^(a, b, c, d)^ in the same column indicate significant differences (*P* < 0.05).

The band assigned to the CH_2_ and CH_3_ bending vibrations was observed near 1450 cm^−1^ ^49,50^. It was reported that the Raman intensity of aliphatic amino acids decreased with the exposure of aliphatic residues^16^. In this study, ultrasound and high-pressure homogenization significantly decreased the intensity of Raman bands corresponding to CH_2_ and CH_3_ bending vibrations, which suggested that aliphatic amino acids tended to be exposed to the polar aqueous solvent^50^. The bending intensity of the SP aliphatic amino acid increased during interaction with PC because the fatty amino acids were embedded in the molecules under SP-PC interaction. Ultrasound treatment and high-pressure homogenization did not significantly affect the bending intensity of aliphatic amino acids in the SP-PC complex.

In comparison, ultrasound treatment and high-pressure homogenization exposed more hydrophobic residues to promote hydrophobic interaction between SP and PC, which was beneficial for stabilization of the nanoemulsion. Ultrasound treatment and high-pressure homogenization similarly promoted SP-PC interaction. It could be concluded that SP-PC interaction had a more significant effect on side chain structure than that observed under ultrasound treatment and high-pressure homogenization.

#### Disulfide bond configuration in soybean protein

Disulfide bonding is an important force maintaining protein tertiary structure. The Raman bands located in the range of 500–550 cm^−1^ correspond to disulfide bridges in soybean protein. Proteins and peptides containing cysteine residues usually show a band in the Raman spectrum near 510 cm^−1^ which has been assigned to the S-S stretching vibrations of disulfide bonds in the lowest potential energy conformation, that is, in the *gauche-gauche-gauche* (*g-g-g*) conformation. The bands at 525 and 540 cm^−1^ have been assigned to *gauche-gauche-trans* (*g-g-t*) and *trans-gauche-trans* (*t-g-t*) rotational isomers, respectively^51^. As depicted in Fig. 3, the disulfide bond conformation of SP was mainly located at 516 cm^−1^, which suggested that *g-g-t* was the main disulfide bond conformation in SP^52^. In comparison, ultrasound treatment and high-pressure homogenization did not significantly alter disulfide bond conformation. The Raman spectrum of the disulfide bond of the SP-PC complex system was located in the range of 515–520 cm^−1^, which suggested that SP-PC interaction did not change the disulfide bond configuration^53^.

#### Phosphatidylcholine structure

Two peaks associated with vibrations of PC could be observed in Fig. 3. The peaks at 1057 cm^−1^ and 1249 cm^−1^ correspond to symmetric and asymmetric vibrations of PO^2−^ groups of PC, respectively^54^. The C-C bending vibrations in the Raman spectra could be used to characterize the all-trans chain conformation changes of the PC. The in-phase and out-of-phase skeletal C-C stretching vibrations were located within the 1000–1200 cm^−1^ range^55^. The *I*_1090_/*I*_1129_ ratio implies the combination of intermolecular and interchain disorder. Further, the intensity ratio of 1090/1064 cm^−1^ was used to probe the extent of *trans/gauche* isomerization^56^. In this study, the structure of PC was analyzed by differential spectra to subtract background caused by protein, and the disorder of lipid chains was expressed through *I*_1090_/*I*_1064_ and *I*_1090_/*I*_1129_, as shown in Table 5.

**Table 5:** Normalized intensities of the *I*_1090_/*I*_1064_ and *I*_1090_/*I*_1129_ in phospholipids after different treatments.

Sample	*I*_1090_/*I*_1064_	*I*_1090_/*I*_1129_
D-SP-PC	0.73 ± 0.00^c^	1.28 ± 0.01^c^
U-SP-PC	1.82 ± 0.01^b^	2.24 ± 0.02^b^
H-SP-PC	2.44 ± 0.02^a^	2.66 ± 0.01^a^

Different letters^(a, b, c)^ in the same column indicate significant differences (*P* < 0.05).

As shown in Table 5, both ultrasound treatment and high-pressure homogenization increased the intensity of *I*_1090_/*I*_1064_ and *I*_1090_/*I*_1129_ of PC, indicating that the two treatments increased the hydrocarbon content and the disorder of lipid chains^54^. The interaction between SP and PC was enhanced by ultrasound and high-pressure homogenization, which further proved that the interaction site of SP-PC consisted of a hydrophobic amino acid side chain and phospholipid hydrophobic lipid chain. The stronger SP-PC interaction promoted by high-pressure homogenization could be proven by the higher intensity of *I*_1090_/*I*_1064_ and *I*_1090_/*I*_1129_.

## Conclusions

In summary, the properties of SP-PC nanoemulsions prepared by ultrasound and high-pressure homogenization treatments were investigated in this study. It was found that the high-pressure homogenization produced a more stable nanoemulsion compared to the ultrasound treatment. The Raman spectroscopy results showed that the two treatments increased α-helix and unordered structures of SP but decreased the β-structures. The interactions between soybean protein and phosphatidylcholine significantly decreased the amount of α-helix structure while increasing the unordered and β-sheet structures. The ultrasound and high-pressure homogenization treatments exerted contrary influences on unordered structures and β-turn structures, which was the reason that nanoemulsions prepared by the two treatments exhibited different stability and interaction characteristics. Under ultrasound and high-pressure homogenization, tryptophan and tyrosine residues of soybean protein tended to become exposed, which promoted the hydrophobic interaction between soybean protein and phosphatidylcholine. However, according to the *g-g-t* vibrational mode–the predominant signal corresponding to disulfide bonds in the soybean protein–neither the two preparation methods nor SP-PC interaction significantly changed the conformations of disulfide bonds. The interaction between SP and PC did not change the structure of disulfide bonds. High-pressure homogenization increased the disorder of lipid chains of PC, promoting SP-PC interaction and increasing the stability of nanoemulsions.

## Materials and Methods

### Materials

Soybean protein (SP) with a protein content of 89.21% was obtained from Lanshan Shandong Co., Ltd. (Liao Cheng, Shandong Province, China). Phosphatidylcholine (PC) was purchased from Shanghai Kaiyang Biotechnology Co., Ltd. (Shanghai, China). Sunflower oil was purchased from COFCO Co., Ltd. (Harbin, Heilongjiang Province, China). All other chemicals were of analytical reagent grade.

### Preparation of crude emulsions

SP (1.5%, w/v) was thoroughly mixed with PC in a 10:1 (w/w) ratio before being dispersed in 98.5 mL phosphate buffer solution (0.05 M, pH 7.4). 5 g sunflower oil was then added to the mixture, followed by homogenization at ambient temperature using an Ultra-Turrax T18 homogenizer (ANGNI Co. Ltd., Shanghai, China) at 14,000 rpm for 5 min to obtain the crude D-SP-PC emulsion.

### Ultrasound preparation of SP-PC nanoemulsions

D-SP-PC emulsions were subjected to ultrasound treatment using an ultrasound processor (NingBo Scientz Biotechnology Co. Ltd., Ningbo, Zhejiang Province, China) with a titanium probe (diameter of 3 cm). The ultrasound treatments were performed at an output intensity of 500 W for 9 min with an intermittent time of 5 s. The treatment was carried out in a double-walled cooling water jacket to maintain a constant temperature of 4 °C. The nanoemulsions prepared by ultrasound treatment were labeled as U-SP-PC nanoemulsion.

### High-pressure homogeneous emulsification process of SP-PC nanoemulsions

D-SP-PC emulsions were homogenized using a high-pressure homogenizer (Stansted Fluid Power Ltd., Essex, UK). The emulsions were treated at 100 MPa by the high-pressure homogenizer (flow rate of 10 L/h) outfitted with a high-pressure plastic needle-seat valve at 4 °C. The nanoemulsions prepared by high-pressure homogeneous treatment were labeled as H-SP-PC nanoemulsion.

### Particle size, polydispersity index and zeta (ζ) potential measurements

The particle size and polydispersity index (*PDI*) of the nanoemulsions were quantified using a laser particle-size analyzer (Zetasizer Nano-ZS 90, Malvern Instrument Co., Ltd., Worcestershire, UK) according to the method by Leong *et al*.^57^ To prevent multiple scattering, the nanoemulsions were diluted in sodium phosphate buffer. The refractive index of 1.46 for nanoemulsion particle and 1.33 for water dispersion were chosen. The results were expressed as the volume-weighted mean diameter D_[4,3]_^58^. The ζ*-*potentials of nanoemulsion droplets were evaluated using a zeta potential analyzer (Zetasizer Nano-ZS 90, Malvern Instrument Co., Ltd., Worcestershire, UK). The untreated and treated samples were diluted to an appropriate concentration using deionized water.

### Turbiscan stability index (TSI)

TSI was used to evaluate the stability of the nanoemulsions, particle coalescence and settling processes using the Turbiscan Lab Expert Concentration System Stability Analyzer (France Formulation Company, Toulouse, France). A higher TSI value indicates that the nanoemulsion is less stable. In this study, 18 mL stabilized nanoemulsion was placed in the cylindrical glass of a Turbiscan. The detectors were used to scan along the vertical direction of each nanoemulsion sealed within vials every 30 min for 6 h at 55 °C.

### 3D Confocal laser scanning microscopy

Proteins can be stained by Nile Blue dye, emitting green light. A Leica TCS SP2 confocal laser scanning microscope (Nano Focus, Heidelberg GmbH, Germany) was used for this test. An aliquot of 10 μL of stained nanoemulsion was transferred onto a microscope slide and covered with a coverslip. A He-Ne laser operating at an excitation wavelength of 633 nm was employed to image the proteins. The observations were performed using 40× objectives.

### Optical microscopy

Optical microscopy images were recorded by an XSJ-2 optical microscope (Chongqing, China) to analyze the morphology of nanoemulsions. A drop of nanoemulsion was absorbed on the glass slide, and then the glass slide was covered and observed under a microscope with an objective lens of 40× magnification power.

### Raman spectroscopy analysis

The Raman spectra of all samples were recorded on a Perkin Elmer Raman Station 400 F Dispersive Raman Spectrometer equipped with a 785 nm diode laser. The laser was focused on the samples on glass slides. Each spectrum was obtained under the following conditions: 80 mW of laser power; 10 scans; 60 s exposure time; 2 cm^−1^ resolution; Raman spectra range of 400–2000 cm^−1^. Each sample was scanned at least three times. The mean of the measurements was used to plot the Raman spectra. Errors in band position were within ± 3 cm^−1^.

Spectral data were smoothed, baseline-corrected, and normalized against the phenylalanine (Phe) band at 1003 ± 1 cm^−1^ using Grams 32 software (Galactic Industries Corporation, Salem, NH, USA). The Phe band located near 1003 cm^−1^ was used as the internal standard to normalize the spectra because it is insensitive to the microenvironment, according to other researchers^16^. Assignment of the visible bands to vibrational modes of the peptide backbone or amino acid side chains was carried out by comparing the Raman spectra of the samples with those in the references^59^. Quantitative analysis on the secondary structure of SP under specific conditions was performed by Gaussian fitting using the Peakfit 4.12 software (Seasolve Software, Framingham, MA). Raman spectra (400–2000 cm^−1^) were plotted as relative intensity (arbitrary units) against Raman shift (wavenumber (cm^−1^)).

### Statistical analysis

All experiments were conducted in triplicate, and the results are presented as the mean ± standard deviation. Means were compared using ANOVA followed by Duncan’s test (p < 0.05). Statistical and chart analysis was performed using Origin 9.1 software.

## Supplementary information

Picture description

## Data Availability

All data generated or analysed during this study are included in this published article.
