# Supplementary material for: Stability Mechanism of Two Soybean Protein-Phosphatidylcholine Nanoemulsion Preparation Methods from a Structural Perspective: A Raman Spectroscopy Analysis
Source: Sci Rep. 2019 May 6;9:6985. doi: 10.1038/s41598-019-43439-5 (PMC6502802; doi:10.1038/s41598-019-43439-5)
Supplement: Supplementary file 1 — Picture description [file 41598_2019_43439_MOESM1_ESM.pdf]

Stability Mechanism of Two Soybean Protein-Phosphatidylcholine Nanoemulsion Preparation Methods from a Structural Perspective: A Raman Spectroscopy Analysis

Ying Zhu [1], Yang Li [1, 2], Changling Wu [1], Fei Teng [1], Baokun Qi [1], Xiaonan Zhang [1], Linyi Zhou [1], Guoping Yu [1], Huan Wang [1], Shuang Zhang [1], Zhongjiang Wang [1, *] and Lianzhou Jiang [1, 3, *]

[1] College of Food Science, Key Laboratory of Soybean Biology in Chinese Ministry of Education, Northeast Agricultural University, Harbin 150030, China;

[2] Institute of Food Industry Research in Harbin, Harbin 150030, China;

[3] National Research Center of Soybean Engineering and Technology, Harbin 150030, China

[*] Correspondence: wzjname@126.com; jlz0109@neau.edu.cn

Ying Zhu and Yang Li contributed equally to this work.

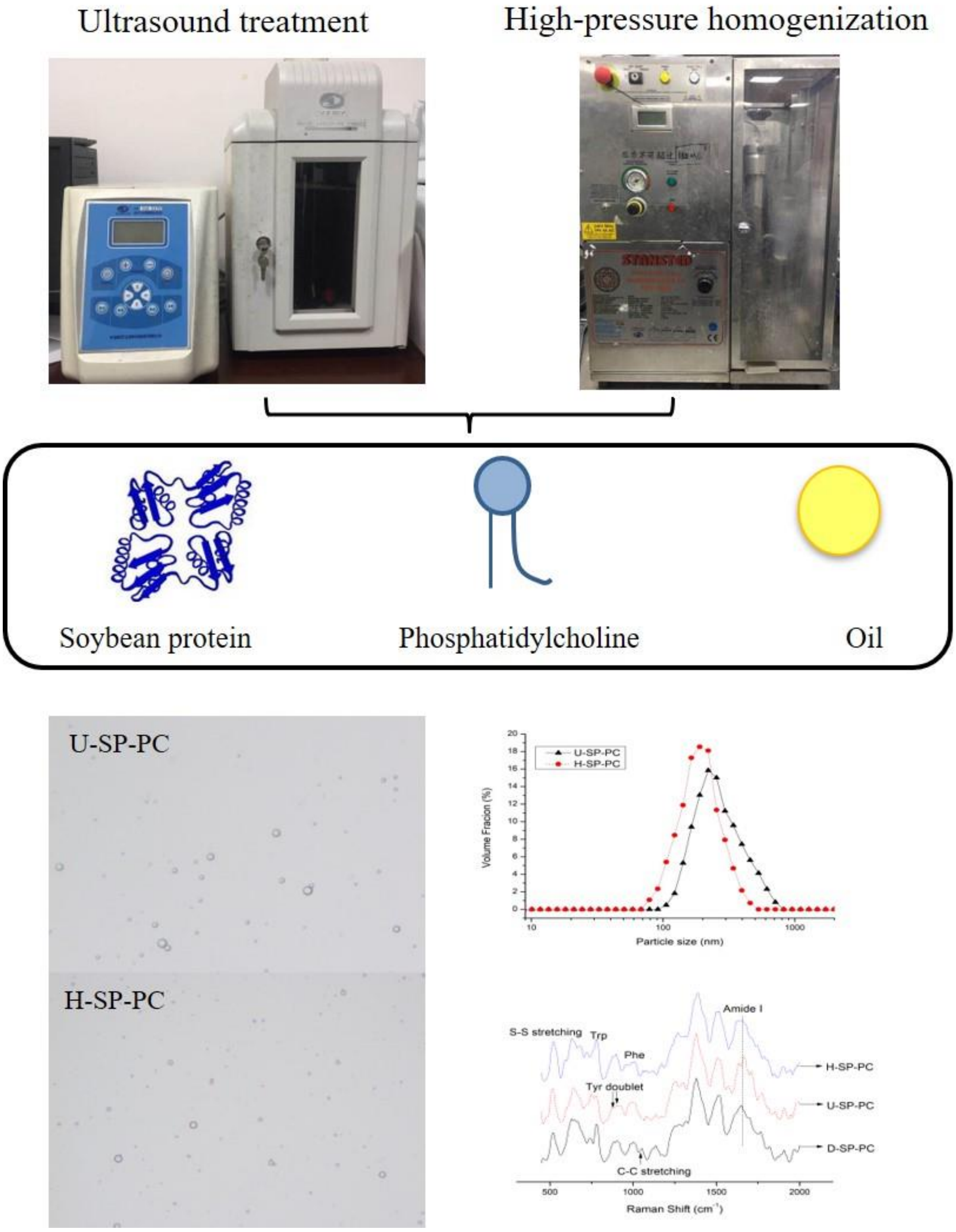
Ultrasound treatment
High-pressure homogenization
Soybean protein
Phosphatidylcholine
Oil
U-SP-PC
H-SP-PC
U-SP-PC
H-SP-PC
Volume Fraction (%)
Particle size (nm)
S-S stretching
Trp
Phe
Tyr doublet
Amide I
C-C stretching
H-SP-PC
U-SP-PC
D-SP-PC
Raman Shift (cm⁻¹)
